# The Impact of the Mediterranean Diet, Physical Activity, and Nutrition Education on Pediatric Metabolic Dysfunction-Associated Steatotic Liver Disease (MASLD): A Review

**DOI:** 10.3390/nu18010028

**Published:** 2025-12-20

**Authors:** Melvin Bernardino, Claudio Tiribelli, Natalia Rosso

**Affiliations:** 1 MASLD Unit, Fondazione Italiana Fegato, 34149 Trieste, Italy; ctliver@fegato.it; 2 Department of Life Sciences, University of Trieste, 34100 Trieste, Italy; 3 Philippine Council for Health Research and Development, Department of Science and Technology, Taguig City 1631, Philippines

**Keywords:** pediatric MASLD, Mediterranean diet, nutrition education, physical activity, nutrition

## Abstract

**Background/Objectives**: Metabolic dysfunction-associated steatotic liver disease (MASLD) is now the most prevalent chronic liver disorder among children and adolescents, mirroring the rise in pediatric obesity. The Mediterranean diet (MD) has demonstrated anti-inflammatory, antioxidant, and beneficial effects on different health outcomes across different life stages. The MD’s effect has been explored in adult MASLD, but there is limited information on the pediatric population. However, evidence on pediatric MASLD should be explored given its rising prevalence. Therefore, the aim of this review is to collect human studies assessing the effect of MD interventions on pediatric MASLD, focusing on key pathophysiological mechanisms. It also examines other interventions, including specific energy/macronutrient prescriptions, nutrition education or counseling, and physical activity components. **Methods**: A comprehensive search of PubMed, Scopus, and Web of Science was conducted using terms related to the Mediterranean diet, nutrition education, physical activity, pediatrics, and MASLD/NAFLD. Pre-determined inclusion and exclusion criteria were used to collect eligible studies to be included in the review. Study quality was assessed using the Academy of Nutrition and Dietetics Quality Criteria Checklist. Screening, data extraction, and appraisal were performed independently, with discrepancies resolved through discussion, and the findings were synthesized qualitatively. **Results**: This review synthesizes findings from eight human studies evaluating the impact of the MD, alone or integrated with structured exercise and nutrition education, on pediatric MASLD. Interventions consistently demonstrated reductions in hepatic steatosis, liver stiffness, and fibrosis markers, alongside improvements in inflammatory cytokines, oxidative stress defenses, and liver enzymes. The MD also enhanced lipid and glycemic profiles, lowering triglycerides, total cholesterol, and insulin resistance indices. Nutrition education and family-centered approaches improved adherence, while structured, enjoyable physical activity enhanced outcomes and long-term sustainability. **Conclusions**: Collectively, the MD, particularly when combined with exercise and tailored education, offers a safe, effective, and comprehensive lifestyle intervention for pediatric MASLD. Nonetheless, current evidence remains limited by small sample sizes, heterogeneity in protocols, and short follow-ups. Larger, multicenter randomized trials with standardized designs are needed to establish best practices and long-term efficacy.

## 1. Introduction

Metabolic dysfunction-associated steatotic liver disease (MASLD) has emerged as the most prevalent chronic liver disorder in children and adolescents worldwide [1,2], paralleling the alarming rise in pediatric obesity [3,4,5,6]. Currently, MASLD affects approximately 38% of the adult population and 7% to 14% of the pediatric population [5]. In the last three decades, there has been an increasing prevalence of MASLD among children, adolescents, and young adults [7]. MASLD is considered a hepatic manifestation of metabolic syndrome due to its close associations with abdominal obesity, insulin resistance, and atherogenic dyslipidemia. Pediatric MASLD is associated with increased cardiometabolic risk, reduced quality of life, and potential progression to advanced liver disease in adulthood [8,9]. Early intervention is crucial, as pediatric-onset MASLD not only carries a higher lifetime risk for complications [10] but is also more responsive to lifestyle-based management than pharmacological approaches [11,12,13,14].

In adult MASLD, practice guidelines and the current literature show that lifestyle and dietary changes are the most effective and safe strategies for prevention and management of MASLD [11,13,15]. In particular, the Mediterranean Diet (MD) has gained substantial attention for its anti-inflammatory, antioxidant, and insulin-sensitizing properties. MD is a dietary pattern traditionally observed in countries bordering the Mediterranean Sea. It is characterized by high consumption of vegetables, fruits and nuts, legumes, and unprocessed cereals; low consumption of meat and meat products; and low consumption of dairy products [16,17,18]. The health-promoting effects of the MD are largely attributed to the synergistic actions of its nutrient-dense components, which are rich in dietary fiber, polyphenols, antioxidants, vitamins, minerals, monounsaturated acids (MUFAs), and polyunsaturated fatty acids (PUFAs) [19].

Furthermore, in adult MASLD research, the combination of MD adherence with regular physical activity [20,21,22,23], often reinforced through structured nutrition education [11,24], has consistently demonstrated additive benefits in reducing hepatic steatosis and improving metabolic outcomes [25]. Research has shown that nutrition education interventions significantly impact patients’ knowledge, attitudes, and behaviors toward food and nutrition. However, in the pediatric population, there is limited knowledge to follow about specific dietary patterns and dietary composition, and their combined effect on pediatric MASLD is unclear regarding nutrition education and physical activity specifically.

The aim of this review is to collect human studies assessing the effect of MD interventions on pediatric MASLD by extracting data on the effects on hepatic steatosis, inflammation markers, oxidative stress markers, liver parameters, the blood lipid profile, the blood glucose profile, and anthropometric measures. Additionally, this review also extracted data on whether the studies incorporated specific dietary prescriptions on energy and macronutrient distribution, nutrition education/counseling, and physical activity. This review systematically examined patterns across human studies to clarify the consistency of benefits, identify gaps in the literature, and provide practical insights for healthcare professionals designing lifestyle interventions for pediatric MASLD.

## 2. Materials and Methods

### 2.1. Literature Search Strategy

A comprehensive literature search was conducted to identify human studies assessing the effects of the MD pattern. Electronic databases, including PubMed, Scopus, and Cochrane, were searched, using terms and keywords related to “Mediterranean diet,” “nutrition education,” “physical activity,” “pediatric,” “MASLD,” and “NAFLD.” Boolean operators (AND, OR) were applied to combine search terms appropriately. In this review, the term MASLD was used according to the updated nomenclature. However, it is acknowledged that the findings also apply to NAFLD, as this was the term predominantly used in the existing literature. No date restrictions were applied in the selection of studies, and no specific geographical limitations were imposed.

### 2.2. Inclusion and Exclusion Criteria

Inclusion criteria were (a) original human studies (randomized controlled trials, quasi-experimental studies, cross sectionals—analytical or observational designs); (b) participants aged 0–18 years diagnosed with MASLD/NAFLD; (c) interventions incorporating the Mediterranean dietary pattern; and (d) reporting of liver-related outcomes such as hepatic steatosis, inflammation, oxidative stress, liver enzymes, lipid or glycemic profiles, and anthropometric measures. Exclusion criteria were (a) animal or in vitro studies; (b) interventions without a dietary component based on the Mediterranean pattern; (c) studies of adult populations; and (d) conference abstracts, reviews, or editorials without primary data.

### 2.3. Quality Assessment

The titles and abstracts of the identified records were screened for relevance, followed by full-text review for eligibility. The quality of the retrieved articles was evaluated using the Academy of Nutrition and Dietetics Quality Criteria Checklist (ANDQCC) for primary research. [26]. This checklist comprises ten questions assessing validity. It examines both internal and external biases to appraise the rigor of the studies’ inclusion and exclusion criteria, methods of data collection and analysis, and the applicability of the findings, ultimately grading the overall study quality. The findings were synthesized qualitatively, highlighting areas of emerging evidence, as well as gaps in the existing literature. Discrepancies arising during study selection, data extraction, or quality appraisal were addressed through deliberation among the review team and by referring to the underlying evidence.

## 3. Results

### 3.1. Quality of the Studies

Eight original research articles met the predefined inclusion and exclusion criteria. All examined the MD as a dietary pattern for pediatric populations with MASLD. As shown in Table 1, all studies received a positive quality rating according to the ANDQCC, reflecting a methodological standard for human studies across the evidence base.

This review synthesizes key evidence on the effects of the MD on pediatric MASLD, with particular focus on hepatic steatosis, fibrosis, inflammatory markers, oxidative stress markers, blood glucose regulation, lipid profile, and anthropometric measures. The review also carefully extracted data on each study regarding the incorporation of specific recommendations on energy requirements, macronutrient distribution, the delivery of nutrition education, and the implementation of physical activity.

### 3.2. Effect of the Mediterranean Diet on Liver Health and Metabolic and Anthropometric Outcomes in Pediatric MASLD

This review synthesizes key evidence on the effects of the MD, with particular focus on hepatic steatosis, fibrosis, inflammatory markers, oxidative stress markers, blood glucose regulation, lipid profile, and anthropometric measures, as summarized in Table 2.

Interventions demonstrate improvements in hepatic outcomes, including reductions in hepatic steatosis [27,28,29,30,31,32], liver stiffness [29,32], and fibrosis markers [30,32]. These benefits are often accompanied by decreases in inflammatory and oxidative stress markers, such as tumor necrosis factor-alpha (TNF-α), interleukin-6 (IL-6), malondialdehyde, and C-reactive protein (CRP) [31], suggesting the systemic anti-inflammatory and antioxidant effect of the diet. Liver function parameters, such as aspartate aminotransferase (AST) [27,28,29,30,31,32], alanine aminotransferase (ALT) [27,28,29,31,32], and occasionally gamma-Glutamyl transferase (GGT) [30,31], frequently improve following adherence to the MD, reflecting enhanced hepatic health. Concurrently, favorable changes in blood lipid and glucose profiles, including reductions in triglycerides (TG) [27,28,29,31,35], low-density lipoprotein (LDL) cholesterol [32], and insulin resistance [27,28,29,31,32], highlight the diet’s role in mitigating cardiometabolic risk factors commonly associated with pediatric MASLD. Improvements in anthropometric measures, such as body mass index (BMI) [27,28,29,30,31,32], waist circumference [28,31,32], and body fat [28,29,31], further support the MD’s contribution to obesity management and metabolic control in children. Adherence appears critical to achieving these benefits, with studies indicating that higher compliance, often measured using the Mediterranean Diet Quality Index for children and adolescents (KIDMED), is associated with greater reductions in hepatic fat [33,34] and inflammation [34].

### 3.3. Dietary Composition of Mediterranean Diet Design for Pediatric Population with MASLD

The dietary composition of the MD across pediatric MASLD interventions consistently emphasizes a balanced macronutrient distribution designed to support metabolic health while reducing liver fat accumulation. Most studies have targeted carbohydrate intake at approximately 40–45% of total energy, favoring complex carbohydrates from unrefined sources such as whole grains, legumes, vegetables, and fruits. Fat intake was typically set between 30% and 40% of total calories, with a strong emphasis on unsaturated fats, particularly monounsaturated fats from extra virgin olive oil (EVOO) and nuts, while limiting saturated fat to less than 10%. Protein contributed about 20% of total energy, sourced primarily from fish, legumes, and dairy, with a reduced intake of red and processed meats. Table 3 summarizes the macronutrient distribution (% of total energy) and additional dietary recommendations reported in each study.

Deshmukh et al. (2024) [32] prescribed an MD providing 40–45% carbohydrates, 30–35% fat (with saturated fat under 10%), and 20% protein. This included daily consumption of colorful vegetables, fish (or limited red meat for non-vegetarians), legumes, multigrain atta (flour blend made from combining several different grains), walnuts, and the use of olive or mustard oil. Yurtdaş et al. [31] implemented a similar macronutrient distribution—40% carbohydrates, 35–40% fat (less than 10% saturated fat), and 20% protein—with daily inclusion of fish, legumes, walnuts (20 g/day), and olive oil (30–45 g/day), alongside avoidance of processed foods and sugary drinks. Akbulut et al. [29] reported an MD composed of 35–40% fat (mainly from EVOO), 40–44% carbohydrates, and 20% protein, emphasizing plant-based foods and whole cereals.

Longer-term studies like Pacifico et al. (2013) [28] and Nobili et al. (2006) [27] followed hypocaloric MD with carbohydrates comprising 50–60%, fat 23–30% (with two-thirds from unsaturated fats), and protein 15–20%. These diets stressed unrefined carbohydrates, high fiber intake, and a balanced omega-6-to-omega-3 fatty acid ratio (~4:1), reflecting traditional MD patterns. Fat quality was a recurrent focus, with efforts to replace saturated fats with MUFAs and PUFAs, and specifically highlighted maintaining an omega-6-to-omega-3 ratio close to 4:1 to support anti-inflammatory effects. Across studies, carbohydrate sources are emphasized as unrefined; protein is derived mainly from legumes, fish, and moderate dairy with reduced red and processed meats; and healthy fats predominantly come from EVOO, nuts, and fish.

Overall, the MD interventions adopt a balanced macronutrient composition that neither excessively restricts nor overemphasizes any single nutrient, a pattern likely contributing to the observed improvements in liver health and metabolic parameters in children and adolescents with MASLD. This macronutrient distribution supports weight management, insulin sensitivity, and reduces hepatic fat.

### 3.4. Adherence to Mediterranean Diet

Two cross-sectional studies explored the relationship between MD adherence and MASLD in children and adolescents. Cakir et al. (2016) [33], using the KIDMED index on 106 obese participants, found no significant associations between MD adherence and liver steatosis, liver enzymes, lipids, or insulin resistance. However, higher adherence was linked to lower BMI, suggesting potential benefits for anthropometric outcomes. In contrast, Della Corte et al. (2017) [34] reported that poor MD adherence increased the risk of MASLD, advanced liver fibrosis, insulin resistance, and elevated CRP levels. Higher adherence was independently protective against liver fibrosis, though it was not associated with differences in BMI or waist circumference. Lower adherence also correlated with higher blood pressure, linking poor diet quality to cardiovascular risk factors. This highlights the importance of promoting consistent dietary adherence to achieve long-term hepatic and cardiometabolic benefits in this vulnerable population. This evidence suggests that the MD may play a supportive role in improving cardiometabolic risk factors and liver health in pediatric MASLD, but further well-designed studies are needed to clarify its specific impact on hepatic outcomes. The KIDMED index, particularly as a self-reported tool, reflects “habitual” dietary patterns and may not fully capture actual adherence to the Mediterranean diet. This limitation may indeed contribute to inconsistent findings. However, to the best of our knowledge, the two studies included in our review are the only available cross-sectional investigations specifically examining adherence to the Mediterranean diet in relation to pediatric MASLD.

### 3.5. Mediterranean Diet Combined with Physical Activity and Nutrition Education Within the Intervention

Addressing pediatric MASLD often requires more than dietary modification alone. Accordingly, five studies have evaluated comprehensive lifestyle interventions that combine the MD, physical activity, and nutrition education, with key findings summarized in Table 3.

Across the reviewed studies, physical activity was incorporated in the management of pediatric MASLD, although the intensity, duration, and flexibility of exercise prescriptions varied notably. Deshmukh et al. (2024) [32] adopted a high-intensity interval training (HIIT)-based approach, complemented by additional aerobic activity, such as walking, jogging, and cycling, for 30–60 min per day, at least five times a week. This combined strategy ensured both vigorous and moderate activity exposure, potentially enhancing cardiovascular fitness and metabolic outcomes. In contrast, Yurtdaş et al. (2022) [31] instructed participants to maintain their usual activity levels. This approach was reasonable, as the prescribed total energy intake was based on each participant’s BMI-derived daily energy requirement, incorporating a low physical activity factor. While this control approach is valuable for isolating dietary effects, it limits insights into the potential additive benefits of structured exercise.

Akbulut et al. (2022) [29] implemented a progressive, child-centered aerobic program, starting with 30–45 min, three days per week, and increasing to 60 min for four to five days weekly. Importantly, children could choose enjoyable activities such as football, swimming, and dancing, which may improve adherence. This intervention also actively targeted sedentary behaviors, encouraging lifestyle-integrated physical activity such as active school breaks and stair use, which is an important behavioral change component often overlooked in purely exercise-based prescriptions. Pacifico et al. (2013) [28] recommended moderate daily exercise for 60 min at least five days per week, alongside a reduction in sedentary activities. The integration of exercise within a broader lifestyle program, including family involvement, aligns with evidence that parental support improves compliance in pediatric lifestyle interventions.

Collectively, these findings highlight two main strategies: (a) structured, progressive programs that emphasize enjoyment and lifestyle integration [28,29] and (b) higher-intensity, performance-oriented regimens [32]. Programs that encourage choice, incorporate daily life activities, and address sedentary behavior may yield better long-term adherence than prescriptive or unmonitored regimens.

Nutrition education formed the foundation of all reviewed interventions, with a consistent focus on reducing saturated fat and limiting high-sugar, high-fat processed foods. Deshmukh et al. (2024) [32] provided clear dietary principles restricting saturated fats, processed/packaged foods, alcohol, instant beverages, carbonated/sugary drinks, candy, ice cream, cream biscuits, cakes, noodles, and high-sugar/high-fat sweets. This comprehensive restriction list targeted multiple MASLD risk factors simultaneously, though the success of such stringent advice depends heavily on family support and food availability. Yurtdaş et al. (2022) [31] offered similar dietary restrictions, guided by a dietitian to ensure message consistency across participants. This method may improve the fidelity of nutrition education delivery, but it does not mention portion guidance or food substitutions, which could be key for practical adherence. Akbulut et al. (2022) [29] went further by providing a food-group list with recommended servings, including equicaloric alternatives for restricted foods. This prescriptive approach addresses not only what to avoid but also what to consume, offering a clear pathway for behavior change. The requirement to consume only recommended foods may enhance dietary control but could risk lower adherence in less motivated families.

Pacifico et al. (2013) [28] integrated nutrition education into a family-centered approach, recognizing that parental knowledge and support are vital for sustaining dietary changes in children. While the specifics of the nutrition advice were less detailed in the summary, the family involvement model is strongly supported by pediatric nutrition literature [36,37,38,39,40,41]. Overall, the nutrition education strategies converge on similar dietary principles but differ in delivery method, such as providing dietitian-led, family-centered, highly prescriptive, and practical tools (food lists; food substitutions). Interventions that balance restriction with practical, culturally appropriate alternatives and engage families may be more sustainable for pediatric MASLD management.

**Figure 1:**
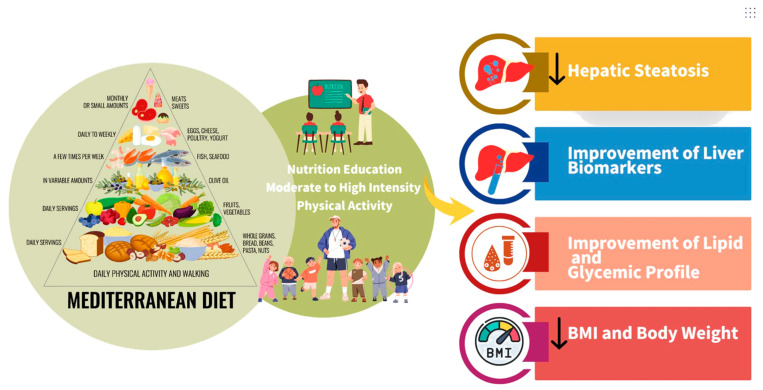
Benefits of adherence to the Mediterranean diet, in combination with nutrition education and moderate-to-high intensity physical activity, regarding hepatic steatosis reduction; improvement of liver function biomarkers and lipid and glycemic profiles; and decreases in body weight and body mass index ↓ decrease. Created with canva.com.

Figure 1 illustrates the benefits of adherence to the MD, combined with nutrition education and moderate-to-high intensity physical activity, demonstrating its role in reducing hepatic steatosis, improving liver function biomarkers, enhancing lipid and glycemic profiles, and lowering body weight and body mass index. This review highlights that integrating MD principles with structured physical activity and tailored nutrition education offers a comprehensive strategy for improving clinical outcomes in pediatric MASLD, with program flexibility, family involvement, and practical dietary guidance emerging as key factors for long-term success.

## 4. Discussion

### 4.1. Mediterranean Diet and Pediatric MASLD

The pathogenesis of pediatric MASLD remains unclear, but evidence suggests that obesity, nutrition, lifestyle variables, and genetic and epigenetic factors may be causally involved in the development of this metabolic liver disease [41]. The 2017 North American Society for Pediatric Gastroenterology, Hepatology, and Nutrition (NASPGHAN) Clinical Practice Guideline for the Diagnosis and Treatment of Nonalcoholic Fatty Liver Disease in Children [12] recommends lifestyle modifications aimed at improving diet quality and increasing physical activity as the first-line treatment for pediatric patients with MASLD. In their expert opinion, the guidelines did not specify a particular dietary pattern, given the lack of strong evidence favoring one approach over another. However, more recent studies and the literature highlight the growing interest in the MD, particularly in the context of childhood obesity, suggesting its potential as a dietary pattern that could be applied to children and adolescents with MASLD.

The MD has been associated with a wide range of health benefits. Evidence indicates a positive association between adherence to the MD and positive health-related quality of life for the pediatric population [42]. Moreover, adherence to the MD was positively associated with higher levels of physical activity, better physical fitness, and lower sedentary behavior [43]. A meta-analysis of 15 randomized controlled trials evaluating MD-based interventions in the pediatric population demonstrated a significant reduction in body mass index and obesity prevalence among children and adolescents aged 3–18 years. According to this pool of RCTs, MD-based interventions in children and adolescents with excess weight appear to be both effective, significantly improving anthropometric outcomes, and safe, with no serious adverse events reported, underscoring their potential as a valuable strategy to help address pediatric obesity [44]. MD may be a suitable dietary pattern for the management of MASLD in pediatric populations. Its applicability is supported by its relevance in young children with obesity and its association with obesity severity [45]. In the context of MASLD, although the MD shows promise in improving liver and metabolic outcomes in pediatric MASLD, adherence remains a major challenge. Children and adolescents may struggle with dietary compliance due to cultural food preferences, the limited availability of traditional MD foods, family eating habits, and the pervasive influence of Westernized diets rich in processed foods and sugar-sweetened beverages. Additionally, adherence can be influenced by socioeconomic status, parental support, nutrition education, and the practicality of implementing dietary changes in daily life. These barriers highlight the importance of structured dietary counseling, family-based interventions, and school/community support to enhance compliance and sustain long-term benefits.

### 4.2. Biological Mechanism of Mediterranean Diet and Its Benefits for Pediatric MASLD

The traditional MD is characterized by a diverse array of minimally processed, fiber-rich plant foods that are abundant in vitamins, minerals, and phytochemicals [46,47]. In the current literature, the potential metabolic and molecular mechanisms mediating the effects of the MD on human health have been explored.

Aside from weight reduction benefits, the MD possesses established health benefits, such as lipid-lowering effects, antioxidant and anti-inflammatory effects, and gut microbiota modulation [48]. The MD’s low saturated fat content, combined with MUFAs and PUFAs from EVOO and nuts, improves lipid profiles, lowers LDL cholesterol, and reduces cardiovascular and MASLD risk [49,50,51,52,53,54,55,56]. Due to the fact that Mediterranean diet components are rich in polyphenols, MUFAs, and PUFAs, particularly from EVOO and fish, the MD reduces oxidative stress and downregulates pro-inflammatory cytokines while enhancing anti-inflammatory mediators, mitigating hepatic inflammation and progression from steatosis to MASH and fibrosis [56,57,58,59,60,61,62,63,64,65]. The MD promotes a diverse, beneficial gut microbiota, increasing short-chain fatty acid production, improving gut barrier integrity, modulating glucose and lipid metabolism, and reducing hepatic inflammation [66,67,68].

**Figure 2:**
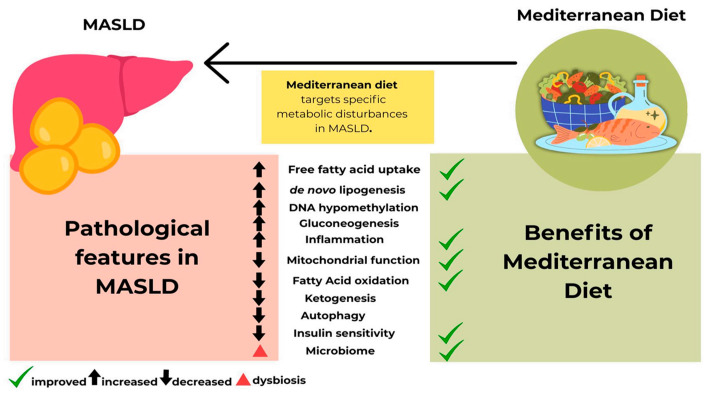
Mediterranean diet targets specific metabolic disturbances of metabolic dysfunction-associated steatotic liver disease (MASLD).

Figure 2 shows that the MD counteracts the key metabolic disturbances that MASLD causes. While MASLD is characterized by increased fat accumulation, impaired mitochondrial activity, higher oxidative stress, inflammation, and reduced insulin sensitivity, the MD works in the opposite direction, such as by improving insulin sensitivity, lowering liver fat production, reducing inflammation and oxidative damage, and supporting healthier gut microbiota. Together, these changes help restore metabolic balance and protect the liver from progressing to more advanced liver problems.

### 4.3. Energy and Macronutrient Distribution in Pediatric MASLD

Optimal growth requires a well-balanced diet that ensures adequate intake of both macronutrients and micronutrients. Macronutrients, which are consumed in the largest quantities, are primarily classified as carbohydrates, proteins, and fats [69]. Adequate nutrition ensures physiological growth while preventing diseases [70]. In the context of pediatric MASLD, this review extracted data on energy requirements and macronutrient distribution prescribed in studies specifically involving children and adolescents with MASLD. This approach provides quantitative targets while also emphasizing the importance of both diet quality and quantity. Table 4 shows an important comparison between the World Health Organization (WHO) recommendation on macronutrients in children vs. on approximation of the human studies included in this review.

### 4.4. Nutrition Education and Physical Activity and Their Benefits for Pediatric MASLD

The incorporation of nutrition education and physical activity with the MD pattern provides benefits for pediatric MASLD. Combined interventions not only optimize reductions in liver fat and improvements in metabolic parameters but also promote sustainable lifestyle changes. Early nutrition education can establish lifelong dietary habits and prevent progression to more severe liver disease. Sustainable lifestyle change interventions emphasize long-term, culturally appropriate, and environmentally conscious dietary changes [72,73,74] that not only improve pediatric MASLD outcomes but also support overall health across the lifespan.

Additionally, family- and school-based programs enhance engagement and efficacy, emphasizing the importance of a supportive environment in achieving lasting results [75,76,77,78]. Lifestyle interventions targeting dietary habits and physical activity constitute the first-line approach in managing pediatric MASLD. Early, structured, and sustained programs can attenuate hepatic steatosis, improve metabolic health, and establish healthy behaviors that persist into adulthood. In pediatric weight management, healthcare professionals, such as registered dietitian nutritionists (RDNs) and nutrition doctors, should deliver multicomponent interventions that integrate medical nutrition therapy (MNT), physical activity, and behavioral strategies [79,80]. Clinical practice guidelines do not specify a fixed “pounds-per-week” or “percentage body weight” target, as is common in adult obesity management recommendations [81]. This distinction reflects the dynamic nature of pediatric growth, in which children are expected to gain height and develop physiologically, rather than simply lose weight, making the primary goal a gradual shift toward a healthier weight trajectory. Therefore, a multidisciplinary approach represents the optimal strategy for managing pediatric MASLD. Future research should focus on identifying strategies to enhance adherence to the MD in the pediatric population, as increasing evidence suggests that, even without weight loss, the MD reduces liver steatosis and improves insulin sensitivity in insulin-resistant adults with MASLD [82]. Furthermore, strategies incorporating early nutrition education and physical activity as part of medical intervention by integrating an enjoyable and age-appropriate plan should be explored.

### 4.5. Recommendations and Future Perspective

Despite promising synthesis in this review, current evidence on the impact of the Mediterranean dietary pattern, combined with nutrition education and physical activity, on pediatric MASLD remains limited. Most available studies are small-scale, short-term, and heterogeneous in design, with variations in dietary protocols, physical activity regimens, and educational approaches that hinder comparability. Dietary adherence is often assessed through self-reported tools, introducing potential bias, while liver outcomes are commonly evaluated using indirect markers, such as ALT, AST, or ultrasound, rather than more sensitive imaging techniques or validated biomarkers.

Moreover, small sample sizes, lack of diverse populations, and limited long-term follow-ups restrict the generalizability and strength of conclusions. Importantly, the combined nature of interventions makes it difficult to isolate the independent effects of diet, physical activity, and education, while sustaining behavioral changes in children and families remains a challenge.

Future research should prioritize large, multicenter, randomized controlled trials with standardized intervention protocols, objective measures of adherence and liver health, and extended follow-up to capture long-term outcomes. In addition, exploring mechanistic pathways, incorporating family- and school-based strategies, and assessing cost-effectiveness will be critical to inform scalable, sustainable interventions for pediatric MASLD.

## 5. Conclusions

The Mediterranean dietary pattern, particularly when combined with structured physical activity and nutrition education, shows consistent benefits for children and adolescents with MASLD. Evidence from human studies demonstrates improvements in hepatic steatosis, liver enzymes, metabolic markers, and anthropometric outcomes, highlighting the diet’s role in reducing cardiometabolic risk. Family involvement, sustainable lifestyle changes, and culturally tailored education enhance adherence and long-term effectiveness. Improving health outcomes related to pediatric MASLD requires an integrated approach that combines adherence to the Mediterranean diet, regular physical activity, and continued nutrition education. Together, these three pillars work synergistically to improve metabolic health and promote long-term healthy behaviors in children. Although current evidence is promising, larger and longer-term studies are needed to confirm efficacy, establish standardized protocols, and support widespread clinical application in pediatric MASLD management.

## Figures and Tables

**Table 1:** Quality assessment of studies using the Academy of Nutrition and Dietetics Quality Criteria Checklist.

Author and Year of Publication	1	2	3	4	5	6	7	8	9	10	Quality Rating
Nobili et al., 2006 [27]	+	-	-	-	-	+	+	+	+	+	Positive
Pacifico et al., 2013 [28]	+	+	-	-	-	+	+	+	+	+	Positive
Akbulut et al., 2022 [29]	+	+	+	-	-	+	+	+	+	+	Positive
Malecki et al., 2021 [30]	+	+	-	-	-	+	+	+	+	+	Positive
Yurtdaş et al., 2022 [31]	+	+	+	+	-	+	+	+	+	+	Positive
Deshmukh et al., 2024 [32]	+	+	+	-	-	+	+	+	+	+	Positive
Cakir et al., 2016 [33]	+	+	+	§	§	§	+	+	+	+	Positive
Della Corte 2017 [34]	+	+	+	§	§	§	+	+	+	+	Positive

(**+**) Answer to validity question was YES. (**-**) Answer to a validity question was NO. (**§**) Not applicable. Validity Questions: (1) Was the research question clearly stated? (2) Was the selection of study subjects/patients free from bias? (3) Were study groups comparable? (4) Was the method of handling withdrawals described? (5) Was blinding used to prevent introduction of bias? (6) Were intervention/therapeutic regimens/exposure factors or procedures and any comparison(s) described in detail? Were intervening factors described? (7) Were outcomes clearly defined and the measurements valid and reliable? (8) Was the statistical analysis appropriate for the study design and type of outcome indicators? (9) Are conclusions supported by results, with biases and limitations taken into consideration? (10) Is bias due to the study’s funding or sponsorship unlikely? Quality Rating Positive: Indicates that the report has clearly addressed issues of inclusion/exclusion, bias, generalizability, and data collection and analysis. Negative: Indicates that these issues have not been adequately addressed. Neutral: Indicates that the report is neither exceptionally strong nor exceptionally weak.

**Table 2:** Summary of studies on the effects of Mediterranean Diet (MD) on pediatric MASLD.

Study (Author, Year, and Country)	Type and Duration of Study	Participant Characteristics on MD Arm; MASLD Diagnosis	Intervention/ Grouping and Comparator/Control	Effects of Mediterranean Diet			
				*Hepatic Steatosis, Fibrosis, Inflammation, and Oxidative Stress*	*Liver* *Parameters*	*Blood Lipid and Blood Sugar Profile*	*Anthropometric and Clinical Measurements*
Deshmukh et al., 2024 [32]; India	Randomized control trial; 180 days	19 children and adolescents with MASLD; male/female; 8–18 years old; age-specific BMI > 85th percentile overweight and obese; liver biopsy	Mediterranean diet vs. calorie-restricted diet	↓ Hepatic steatosis determined by CAP values ↓ Liver stiffness measurement ↓ PNFI ↔ TNF-a ↔ IL-6	↓ AST ↓ ALT	↓ TC ↓ LDL ↓ HOMA-IR	↓ Body weight ↓ BMI ↓ Triceps skinfold thickness ↓ WC
Yurtdaş et al., 2022 [31]; Turkey	Randomized control trial; 12 weeks	22 adolescents with MASLD and obesity; 11–18 years old; age-specific BMI ≥ 95th percentile; liver ultrasound	Mediterranean diet vs. low-fat diet	↓ Hepatic steatosis 13.6% of the adolescents did not have fatty liver ↑ TAS, ↑ PON-1 ↑ GSH-Px ↑ Glutathione ↓ Malondialdehyde ↓ TNF-a, ↓ IL-6 ↓ IL-8, ↓ IL-1β ↑ IL-10, ↓ CRP levels	↓ AST ↓ ALT ↓ GGT	↔ TG ↔ TC ↔ LDL ↔ HDL ↓ Insulin ↓ HOMA-IR	↓ Body weight ↓ BMI ↓ WC ↔ Fat-free mass ↓ Waist–hip ratio ↓ Body fat
Akbulut et al., 2022 [29]; Turkey	Randomized control trial; 12 weeks	30 children and adolescents with MASLD; male/female; 9–17 years old; age-specific BMI > 85 percentile overweight and obese; liver ultrasound	Mediterranean diet vs. low-fat diet	↓ Hepatic steatosis ↓ Liver stiffness	↓ AST ↓ ALT	↓ HOMA-IR ↓ TC ↓ TG	↓ Body weight ↓ BMI ↓ Body fat ratio
Malecki et al. 2021 [30]; Poland	Prospective consecutive study	49 children and adolescents with MASLD; male/female; 3–16 years old; BMI are normal, overweight, and obese; liver ultrasound	Mediterranean diet; compliant vs. non-compliant	↓ APRI (AST to PLT ratio) for patients who 100% followed the lifestyle modification	↓ AST ↓ GGT for patients who 100% followed the lifestyle modification		↓ BMI for patients who 100% followed the lifestyle modification
Pacifico et al., 2013 [28]; Italy	Prospective interventional cohort study; 1 year	120 children and adolescents with MASLD; male/female; 9–17 years old; age-specific BMI > 95 percentile obese; liver ultrasound and MRI	Mediterranean diet; before and after intervention	↓ Hepatic fat fraction ↓ High-sensitivity C-reactive protein	↓ AST ↓ ALT ↔ GGT	↓ TG ↔ TC ↔ LDL ↔ HDL ↔ non-HDL ↓ HOMA-IR ↔ Fasting Glucose	↓ BMI ↓ WC ↓ Fat mass ↓ Diastolic blood pressure
Nobili et al., 2006 [27]; Italy	Prospective observational study with an interventional component	57 children and adolescents with MASLD; male/female; 3–17 years old; age-specific BMI range of 15.2 to 38.4, mean BMI of 26.3; liver ultrasound and MRI	Low-calorie–Mediterranean diet	↓ Hepatic steatosis/↓ echogenicity, reflecting an improvement (reduction) in hepatic fat accumulation	↓ AST ↓ ALT ↔ GGT	↓ TC ↓ TG ↓ Fasting Glucose ↓ Fasting Insulin ↓ HOMA	↓ BMI ↓ Weight
Cakir et al., 2016 [33]; Turkey	Cross-sectional-association	106 children and adolescents, obese with NAFLD; male/female; average BMI of 30.6; average age of 12 years old; liver ultrasound	Mediterranean diet; adherence comparison between obese children with and without NAFLD and healthy children	No significant difference was found in KIDMED index score between NAFLD patients with grade 1, 2, or 3 hepatic steatosis	No significant correlation was found with ALT	No significant correlation was found with TG, TC, or HOMA-IR	KIDMED index score was negatively correlated with BMI. No significant correlation was found with body fat.
Della Corte et al., 2017 [34]; Italy	Cross-sectional-association	243 children, with 166 patients with fatty liver and 77 without fatty liver; 53 cases of NASH; all were obese/overweight (BMI 28.16 kg/m^2^); ages 10–17 years old); liver ultrasound and liver biopsy	Mediterranean diet; adherence comparison between with fatty liver, without fatty liver, and with NASH	↓ Risk of NASH, less hepatic inflammation, and lower NAFLD activity score (NAS) on high adherence to MD ↓ CRP level on high adherence to MD ↑ KIDMED score is independently associated with lower risk of liver fibrosis in children with NAFLD.	↓ ALT and AST levels in the high MD adherence group	Improved insulin sensitivity ↓ HOMA-IR ↓ fasting glucose ↓ TG	No differences were found for anthropometric parameters (BMI, weight, and waist circumference) between these groups. Negative correlation between the lower values of KIDMED score and blood pressure was observed.

↑ increase; ↓ decrease; ↔ did not change; MASLD: metabolic dysfunction-associated steatotic liver disease; NASH: non-alcoholic steatohepatitis; NAFLD: non-alcoholic fatty liver disease; MD: Mediterranean diet; MRI: magnetic resonance imaging; PNFI: Pediatric NAFLD Fibrosis Index; CAP: controlled attenuation parameter; APRI: aspartate aminotransferase-to-platelet ratio index; KIDMED index: Mediterranean Diet Quality Index for children and adolescents; BMI: body mass index; WC: waist circumference; TG: triglyceride; TC: total cholesterol; LDL: low-density lipoprotein; HDL: high-density lipoprotein; AST: aspartate aminotransferase; ALT: alanine aminotransferase; GGT: gamma-glutamyl transferase; HOMA: homeostatic model assessment; HOMA-IR TNF-α: tumor necrosis factor-alpha; IL-6: interleukin-6; IL-8: interleukin-8; IL-1β: interleukin-1 beta; CRP: C-reactive protein; IL-10: interleukin-10; TAS: total antioxidant status; PON-1: paraoxanase-1; GSH-Px: glutathione peroxidase.

**Table 3:** Summary of Mediterranean diet interventions in pediatric MASLD: macronutrient distribution, nutrition education, and physical activity components.

Study (Author, Year, and Country)	Macronutrient Distribution (% of Total Energy) and Additional Dietary Recommendations	Focused on Nutrition Education/Counseling	Physical Activity Recommended During the Study	Summary of Effects on Health Outcomes
Deshmukh et al., 2024 [32] (India) RCT; 180 days	Calorie Intake: Age- and gender-appropriate energy requirements per day Carbohydrates: 40–45% Fat: 30–35% (<10% saturated fat) Protein: 20% Colorful veggies, fish/less red meat, legumes, multi-grain atta, nuts, olive oil/mustard oil; cinnamon, garlic, pepper added	Dietary principles include restricting saturated fat intake and avoiding processed/packaged products, alcohol, instant beverages, carbonated/sugary drinks, candy, ice cream, cream biscuits, cake, noodles, and sweets high in sugar and fat.	High intensity minimum of 3 sets/day for 5 times per week, along with walking/jogging/cycling for at least 30 min to 1 h.	**Hepatic and Fibrosis:** ↓ hepatic steatosis (CAP), ↓ liver stiffness, ↓ pediatric NAFLD fibrosis index; **Inflammation:** No significant change in TNF-α, IL-6; **Liver Enzymes:** ↓ AST, ALT; **Lipids and Glucose:** ↓ TC, LDL, HOMA-IR; Anthropometrics: ↓ body weight, BMI, triceps skinfold, WC
Yurtdaş et al., 2022 [31] (Turkey) RCT; 12 weeks	Calorie Intake: BMI-based energy requirements per day with a low physical activity factor Carbohydrates: 40% Fat: 35–40% (<10% saturated fat) Protein: 20% Fish, legumes 2–3 times/week; walnuts 20 g/day; olive oil 30–45 g/day daily; restrict saturated fats and processed foods	Dietary principles include restricting saturated fat intake and avoiding processed/packaged products, alcohol, instant beverages, carbonated/sugary drinks, candy, ice cream, cream biscuits, cake, noodles, and sweets high in sugar and fat.	Usual level of physical activity	**Hepatic and Fibrosis:** ↓ hepatic steatosis (13.6% no fatty liver at follow-up); **Oxidative Stress:** ↑ TAS, PON-1, GSH-Px, glutathione; ↓ malondialdehyde; **Inflammation:** ↓ TNF-α, IL-6, IL-8, IL-1β; ↑ IL-10; ↓ CRP; **Liver Enzymes:** ↓ AST, ALT, GGT; **Lipids and Glucose:** no significant change in TG, TC, LDL, HDL; ↓ insulin, HOMA-IR; **Anthropometrics:** ↓ body weight, BMI, WC, WHR, body fat
Akbulut et al., 2022 [29] (Turkey) RCT; 12 weeks	Calorie Intake: Age- and gender-appropriate energy requirement per day using Schofield equation for energy expenditure Carbohydrates: 40–44% Fat: 35–40% (<10% saturated fat) Protein: 20% Rich in plant-based foods, extra virgin olive oil as main added fat	During the education session, children received nutritional recommendations and a food-group list specifying preferred choices and approximate serving numbers and sizes per day, based on dietary modeling. Foods to be avoided were identified, and potential alternatives with equivalent caloric values were provided. Patients were instructed not to consume any foods outside the recommended list.	Twelve-week exercise program for 30–45 min/3 days in the first two weeks and for 60 min/4–5 days in the following weeks.	**Hepatic and Fibrosis:** ↓ hepatic steatosis, liver stiffness. **Liver enzymes:** ↓ AST, ALT; **Lipids and Glucose:** ↓ HOMA-IR, TC, TG; **Anthropometrics:** ↓ body weight, BMI, body fat ratio
Pacifico et al., 2013 [28] (Italy) Prospective interventional. 1 year	Calorie Intake: Hypocaloric: 25–30 kcal/kg/day Carbohydrates: 50–60% Fat: 23–30% (two-thirds unsaturated, one-third saturated) Protein: 15–20% Emphasis on unrefined carbs, fiber (whole grains, vegetables, fruits), low-fat dairy; omega-6–omega-3 ratio approx. 4:1	Guidance on healthy eating for child and family.	Moderate daily exercise program (60 min/day at least 5 days a week).	**Hepatic and Fibrosis:** ↓ hepatic fat fraction, ↓ hs-CRP; **Liver enzymes:** ↓ AST, ALT, no change GGT; **Lipids & Glucose:** ↓ TG, HOMA-IR, no significant change in TC, LDL, HDL, fasting glucose; **Anthropometrics:** ↓ BMI, WC, fat mass, diastolic BP
Nobili et al., 2006 [27] (Italy) Prospective observational; interventional	Calorie Intake: Balanced low-calorie: 25–30 kcal/kg/day Carbohydrates: 50–60% Fat: 23–30% (two-thirds unsaturated, one-third saturated) Protein: 15–20% Balanced diet tailored individually; omega-6–omega-3 ratio approx. 4:1; goal of negative calorie balance	Guidance on healthy eating for child and family.	Aerobic exercise (30–45 min/d at least 3 times a week)	**Hepatic and Fibrosis:** ↓ hepatic steatosis; **Liver enzymes:** ↓ AST, ALT, no change GGT; **Lipids and Glucose:** ↓ TC, TG, fasting glucose, insulin, HOMA; **Anthropometrics:** ↓ BMI, weight

↑ increase; ↓ decrease; ALT: alanine aminotransferase; AST: aspartate aminotransferase; BP: blood pressure; BMI: body mass index; CAP: controlled attenuation parameter; CRP: C-reactive protein; GGT: gamma-glutamyl transferase; GSH-Px: glutathione peroxidase; HDL: high-density lipoprotein cholesterol; HOMA-IR: homeostatic model assessment for insulin resistance; IL-1β: interleukin-1 beta; IL-6: interleukin-6; IL-8: interleukin-8; IL-10: Interleukin-10; LDL: low-density lipoprotein cholesterol; NAFLD: non-alcoholic fatty liver disease; PON-1: paraoxonase-1; RCT: randomized controlled Trial; TAS: total antioxidant status; TC: total cholesterol; TG: triglyceride; TNF-α: tumor necrosis factor-alpha; WC: waist circumference; WHR: waist-to-hip ratio.

**Table 4:** Macronutrient distribution: WHO recommendations compared with evidence from Mediterranean diet studies.

Macronutrient	WHO Recommendation [71]	MASLD Studies (Practical Targets) [27,28,29,31,32]
Carbohydrates	45–60% of total energy Free sugars < 10% (ideally < 5%) Mostly from whole grains, fruits, vegetables, pulses	Approximately 40–45% of total energy, prioritizing complex carbs (whole grains, legumes, vegetables, fruits)
Fat	≤30% of total energy Saturated fat < 10%	Approximately 30–40% of total energy, emphasizing unsaturated fats (EVOO, nuts) Saturated fat < 10%
Protein	0.8–0.9 g/kg/day (≈10–15% of energy)	Approximately 20% of total energy, mainly from fish, legumes, dairy; limited red/processed meats

WHO: World Health Organization.

## Data Availability

No new data were created or analyzed in this study. Data sharing is not applicable to this article.

## Abbreviations

The following abbreviations are used in this manuscript:

ALT	Alanine Aminotransferase
ANDQCC	Academy of Nutrition and Dietetics Quality Criteria Checklist
APRI	Aspartate Aminotransferase to Platelet Ratio Index
AST	Aspartate Aminotransferase
BMI	Body Mass Index
BP	Blood Pressure
CAP	Controlled Attenuation Parameter
CRP	C-reactive Protein
EVOO	Extra Virgin Olive Oil
GGT	Gamma-Glutamyl Transferase
GSH-Px	Glutathione Peroxidase
HDL	High-Density Lipoprotein Cholesterol
HIIT	High-Intensity Interval Training
HOMA/HOMA-IR	Homeostatic Model Assessment/Homeostatic Model Assessment for Insulin Resistance
IL-1β	Interleukin-1 Beta
IL-6	Interleukin-6
IL-8	Interleukin-8
IL-10	Interleukin-10
KIDMED	Mediterranean Diet Quality Index for Children and Adolescents
LDL	Low-Density Lipoprotein Cholesterol
MASLD	Metabolic Dysfunction-Associated Steatotic Liver Disease
MASH	Metabolic Dysfunction-Associated Steatohepatitis
MD	Mediterranean Diet
MRI	Magnetic Resonance Imaging
MNT	Medical Nutrition Therapy
MUFA	Monounsaturated Fatty Acid
NAFLD	Non-Alcoholic Fatty Liver Disease
NASH	Non-Alcoholic Steatohepatitis
NASPGHAN	North American Society for Pediatric Gastroenterology, Hepatology, and Nutrition
PNFI	Pediatric NAFLD Fibrosis Index
PUFA	Polyunsaturated Fatty Acid
RCT	Randomized Controlled Trial
TAS	Total Antioxidant Status
TC	Total Cholesterol
TG	Triglycerides
TNF-α	Tumor Necrosis Factor-Alpha
WC	Waist Circumference
WHO	World Health Organization
WHR	Waist-to-Hip Ratio
